# blend4php: a PHP API for galaxy

**DOI:** 10.1093/database/baw154

**Published:** 2017-01-10

**Authors:** Connor Wytko, Brian Soto, Stephen P. Ficklin

**Affiliations:** 1 Department of Horticulture and; 2 School of Electrical Engineering and Computer Science, Washington State University, Pullman, WA 99164, USA

## Abstract

Galaxy is a popular framework for execution of complex analytical pipelines typically for large data sets, and is a commonly used for (but not limited to) genomic, genetic and related biological analysis. It provides a web front-end and integrates with high performance computing resources. Here we report the development of the blend4php library that wraps Galaxy’s RESTful API into a PHP-based library. PHP-based web applications can use blend4php to automate execution, monitoring and management of a remote Galaxy server, including its users, workflows, jobs and more. The blend4php library was specifically developed for the integration of Galaxy with Tripal, the open-source toolkit for the creation of online genomic and genetic web sites. However, it was designed as an independent library for use by any application, and is freely available under version 3 of the GNU Lesser General Public License (LPGL v3.0) at https://github.com/galaxyproject/blend4php.

**Database URL:** https://github.com/galaxyproject/blend4php

## Introduction

Galaxy (1) is an open-source framework designed to facilitate creation and execution of analytical scientific workflows for high-throughput datasets. It also serves as a data integration platform into which users can upload files, manipulate them and perform analytics. Galaxy is self-described as a ‘web-based platform for data intensive biomedical research’ and is used ubiquitously throughout the biological sciences. Moreover, because of its robust support for new tool integration, it can be used by any scientific domain. One advantage with Galaxy is that workflows and integrated tools can be shared with others, allowing new Galaxy instances to be quickly installed for local institutional projects. Additionally, Galaxy maintains analysis history and provides a publishable provenance record that facilitates reproduction of published results.

Galaxy is intended to ease the burden for scientists and informaticians managing large datasets through complex workflows. It also provides resources for software and web developers to create new tools that interact with Galaxy, and, it provides web services via a representational state transfer (REST) application programming interface (API). Using this API, a software tool can programmatically manage users, installed tools, upload and download files, build and execute workflows and more. Some existing tools have been created using this API such as BioBlend (2) which wraps the RESTful API of Galaxy into a native set of functions in the programming language Python. BioBlend was later extended to include BioBlend.objects—an object-oriented interface to BioBlend (3). It was used for the development of the BioMAJ2Galaxy (4) tool which integrates BioMAJ (5) with Galaxy, and is used for development of automated pipelines such as for pancreatic cancer research (6). For Java developers, the blend4j (https://github.com/jmchilton/blend4j) framework provides a similar interface to that of BioBlend.

Here, we report the availability of the blend4php library, a wrapper for the Galaxy API similar to BioBlend. The blend4php library is intended to provide a common framework for simplified access to a Galaxy server for the PHP programming language. The initial audience for the blend4php library is the international group of developers working with Tripal (7), a PHP-based open-source toolkit for the construction of online genomic, genetic and biological databases. Since 2009, Tripal has been adopted by many online biological websites that provide genomic, genetic, breeding and systems-level data to their respective research communities. These so-called community databases also provide analytical tools to their users. Some Tripal-based sites include the Genome Database for Rosaceae (8), KnowPulse (9), the Legume Information System (10), i5K Workspace (11), the Banana Genome Hub (12), CottonGen (13), GeneNetEngine (14) and others. With the advent of next-generation sequencing technologies and the increased quantities of data, integration of Tripal with Galaxy provides an important link for these research communities. Although initially intended as a tool for integration of Tripal and Galaxy, the blend4php library has been developed as an independent project with the expectation that it can be useful for other applications.

## Methods

The blend4php library is implemented in PHP and provides a wrapper (or bindings) to the Galaxy RESTful API. The library requires installation of cURL and PHP-cURL. When possible, the blend4php attempts to provide a one-to-one mapping of PHP functions to Galaxy API functions. This is to ensure transparency between the two such that if issues arise, site developers can more easily translate functions in their PHP code to the respective Galaxy API functions. Additionally, the blend4php library includes unit testing following the PHPUnit testing framework.

Currently, the blend4php library implements 21 Galaxy API classes (in alphabetical order): datasets, data types, folder contents, folders, genomes, group roles, groups, group users, histories, history contents, jobs, libraries, library contents, requests, roles, search, tools, toolshed repositories, users, visualizations and workflows. These classes provide the bulk of the functions needed to manage a remote Galaxy server.

**Figure 1:**
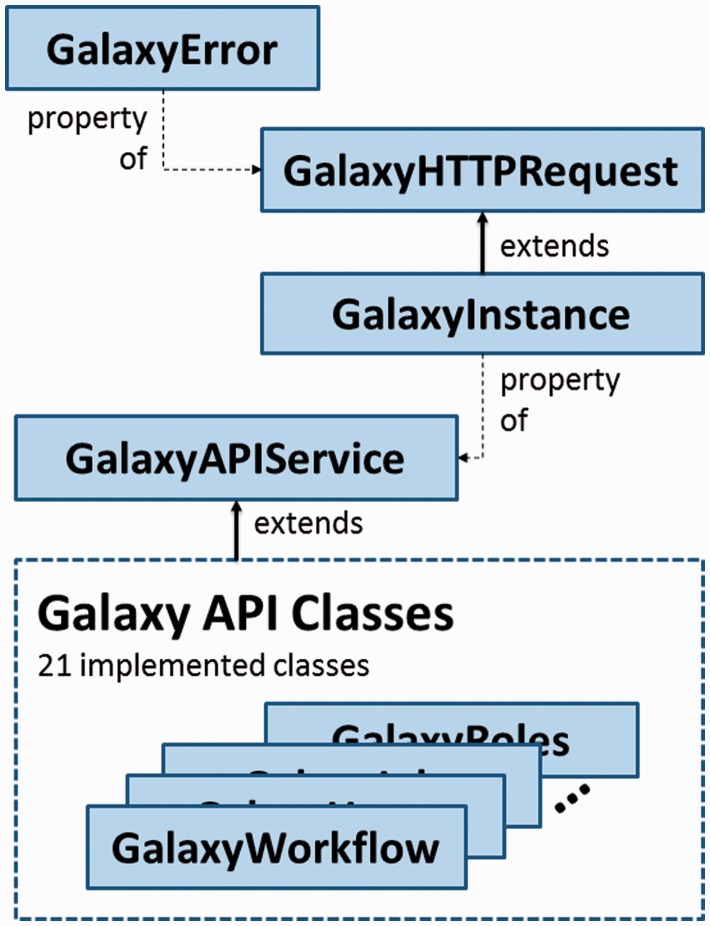
The hierarchical relationships between classes in the blend4php library.

Figure 1 provides an overview of the blend4php code design. The GalaxyError class provides consistent error handling for the library. The GalaxyHTTPRequest class provides functions for connectivity to Galaxy including support for HTTP methods (GET, POST, PUT, PATCH, DELETE) and file uploads. It provides error handling (using the GalaxyError class) and automatic conversion of Galaxy JSON responses to native PHP data types. The GalaxyInstance class extends the GalaxyHTTPRequest and provides authentication and API management functions for Galaxy. The GalaxyAPIService class serves as a base class for all others (e.g. GalaxyHistories, GalaxyGroups, GalaxyUsers etc.). These other classes extend GalaxyAPIService to provide one-to-one mappings to Galaxy API classes. Each GalaxyAPIService child class receives a GalaxyInstance object in its constructor connecting the object with a remote Galaxy instance.

## Results

Currently, blend4php is ready for development of PHP applications. A use case is the Galaxy module for Tripal (named Tripal Galaxy) which is under development. Once completed, it will be possible to download and install this module on any Tripal site to allow a community database to provide analytical tools via Galaxy while maintaining a familiar look-and-feel. The blend4php library also contains a set of unit tests to ensure stability of the library and compatibility with future updates to Galaxy.

Developers can use the blend4php with three steps:

- Instantiate a GalaxyInstance object.
- Use the GalaxyInstance object to authenticate with a remote Galaxy server using credentials of an existing user on the remote server.
- Use any of the other blend4php class functions to interact with Galaxy by providing appropriate parameters.

Online API documentation with example code is available at the blend4php project website.
